# Genetic Assessment of a Captive Population of Eurasian Stone-Curlew (*Burhinus oedicnemus*), Source for the Reinforcement of Wild Populations

**DOI:** 10.3390/biology13120982

**Published:** 2024-11-27

**Authors:** Loïc Lesobre, Alessia Ostolani, Hiba Abi Hussein, Dimitri Giunchi, Mohamed Aourir, Yassine Teyar, Mariella Baratti

**Affiliations:** 1 Reneco International Wildlife Consultants LLC, Al Reem Island, Abu Dhabi P.O. Box 61741, United Arab Emirates; alessia.ostolani@gmail.com (A.O.); habihussein@reneco.org (H.A.H.); 2 Department of Biology, University of Pisa, Via Volta 6, 56126 Pisa, Italy; dimitri.giunchi@unipi.it; 3 Département de Biologie, Faculté des Sciences, Université Ibn Zohr, Agadir BP 8106, Morocco; maourir@gmail.com (M.A.); yassine.teyar@edu.uiz.ac.ma (Y.T.); 4 Research Institute on Terrestrial Ecosystems IRET-CNR, Via Madonna del Piano 10, 50019 Sesto Fiorentino, Italy; mariella.baratti@cnr.it

**Keywords:** ex situ conservation, genetic management, NADH2, microsatellites, population reinforcement, stone-curlew

## Abstract

Although ex situ conservation programs are increasingly valuable support tools for in situ conservation measures, success depends on these captive individuals to be genetically representative of the recipient population. The Eurasian stone-curlew (*Burhinus oedicnemus*) inhabit steppes that represent some of the most degraded and exploited habitats worldwide. A captive breeding program was implemented in Morocco as a pre-emptive effort for the conservation of the North African subspecies *Burhinus oedicnemus saharae*. However, the genetic origins of the founders of the captive flock were unknown. We applied a multi-locus approach to characterize the genetic ancestry of the current captive breeding flock by comparing it to wild populations from both Western and Eastern Morocco. Mitochondrial DNA and microsatellite markers were employed to assess levels of genetic diversity and relatedness within each sample, as well as potential genetic differentiation between wild and captive samples through PCA and admixture analyses. We recovered similar genetic diversity estimates, low levels of relatedness, and little differentiation between captive and wild samples. These results confirmed the Moroccan origin of the founders. We provide recommendations for the optimization of the Eurasian Stone Curlew conservation breeding program but also for future conservation breeding programs to ensure the effective conservation of genetic diversity and wild populations.

## Simple Summary

Captive breeding programs are increasingly valuable for supporting conservation efforts, provided that captive individuals are genetically similar to their wild counterparts. The North African subspecies of the declining Eurasian stone-curlew, which inhabits threatened steppe habitats, is supported by a captive breeding program in Morocco. To assess the origin of the captive birds and their genetic diversity, we compared captive with wild Moroccan stone-curlews using various molecular markers. We found that the captive birds exhibited low relatedness and genetic diversity similar to the wild populations, from which they differed only by marginal genetic differences. This confirmed the Moroccan origins of captive birds as well as their suitability for providing individuals for the release and reinforcement of wild populations. Recommendations were provided to enhance the breeding program’s effectiveness in preserving genetic diversity and supporting wild populations.

## 1. Introduction

As part of the One Plan approach [1,2,3], ex situ Conservation Breeding Programs are recognized as extremely valuable in supporting efficient in situ conservation actions [4]. Their combined use is recognized as a more efficient strategy than using either one of them [5]. Thus, ex situ populations offer valuable resources to conservation efforts, serving as insurance against stochastic events in the wild, providing individuals for conservation translocations, and facilitating both fundamental and applied research aimed at improving the survival of wild populations. Ex situ conservation is thus crucial to ensure the survival of declining species and reinforce populations depleted by human pressure [6,7,8]. Both demographic and genetic features are to be considered in order to ensure viable and healthy populations and to prevent loss of genetic diversity [9,10,11,12,13]. These genetic goals are fundamental and require that captive populations are under strict genetic management to prevent loss of genetic diversity [9,10]. Indeed, ex situ conservation can be associated with genetic changes that can affect the eco-evolutionary trajectories of populations [14], altering their evolutionary potential and their capacity to adapt to environmental changes [15,16]. These potential changes include a reduction of genetic diversity through genetic drift [9], inbreeding depression [17], adaptation to captivity [18], relaxed selection in small populations [19], and loss of local adaptation through outbreeding [20,21]. The first four concerns can be addressed through pedigree management of captive populations [8,10], while the latter requires that founders from the captive population are genetically as close as possible to recipient populations [22]. In addition, a fundamental premise in pedigree management is that founders are unrelated while the variance in relatedness among them is nearly zero [23,24,25].

The Eurasian stone-curlew (*Burhinus oedicnemus*, Linnaeus, 1758) is the northernmost species of the Burhinidae family with a very large range from Western Europe and North Africa to Central Asia and the Middle East [26]. Five subspecies are currently described [26]. It mainly inhabits (pseudo) steppe and agricultural lands that represent some of the most degraded and exploited habitats in the world [27]. Consequently, and despite presenting a globally large distribution, as many steppe species, they have small resident populations isolated in small remnants of suitable habitat, which are often distantly located from one another [28] and show decreasing population trends. In Europe, the nominate subspecies (*B. o. oedicnemus*) suffered a rapid and important population decline over the second half of the last century, which also led to its disappearance as a breeder from the Netherlands, Germany, Slovakia, and Belarus and to its extinction in the Czech Republic and in Slovenia in the 1980s [29]. The main causes of its decline could be attributed to the transformation and reduction of its habitats due to the intensification of agricultural practices, the reduction of grazing activities, the urbanization of rural areas with relative anthropic disturbance, and the widespread use of pesticides [30,31]. The subspecies *B. o. saharae* is widely distributed in Morocco [32] with a consistency estimated at approximately 10,000–100,000 individuals [30]. Its status is, however, not well assessed due to the substantial lack of monitoring programs [33]. In several areas of North Africa, including Morocco, a massive conversion of the natural landscape and rural areas with traditional agricultural practices to irrigated farmland is taking place [34,35]. This habitat transformation may have far-reaching consequences for steppe birds like those already recorded in Europe. Furthermore, in some parts of its range, the species is also subject to hunting, including falconry [36,37,38].

In 2012, the IFHC (International Fund for Houbara Conservation) commissioned Reneco International Wildlife Consultant to develop a preventive strategy for the conservation of the Eurasian stone-curlew in Morocco (*B. o. saharae*). This led to the implementation of a conservation breeding program managed by the ECWP (Emirates Centre for Wildlife Propagation) with the ultimate objective to supplement wild populations in Eastern Morocco. The facility is situated at Enjil (Morocco) and is included in a network of specialized stations for Conservation breeding projects [39]. In 2013, 489 individuals were received from a breeding facility located near Rabat in Morocco. The number of founding individuals, pairing management, and associated levels of relatedness within the population were not documented. However, empirical and simulation studies demonstrated that minimizing mean kinship by selectively breeding individuals descended from underrepresented founders was an effective method for maximizing genome-wide variation, gene diversity, and allelic diversity within captive populations [8,16,40,41]. Therefore, once at ECWP, and to reduce the risk of genetic diversity losses and adaptation to captivity [42], the genetic management strategy implemented focused on three key principles: minimization of the mean kinship within the captive population, inbreeding avoidance, and equalizing family sizes. In addition, birds were housed in pairs, which allowed for clear pedigree records, and chicks were produced through natural reproduction.

With this study, we aimed to investigate the genetic characteristics (i.e., genetic diversity and relatedness levels) of the captive population and compare captive and wild populations from Morocco to confirm the genetic compatibility of captive stock with native Moroccan populations. Furthermore, we investigated the origin of the founders and their level of relatedness. To do so, we applied a multi-locus approach, using both mitochondrial (i.e., NADH2 gene) and nuclear markers (i.e., microsatellites). The resulting data was used to provide recommendations to optimize the genetic management strategy of the conservation program and ensure the efficient conservation of wild populations.

## 2. Materials and Methods

### 2.1. Sample Collection

**Figure 1:**
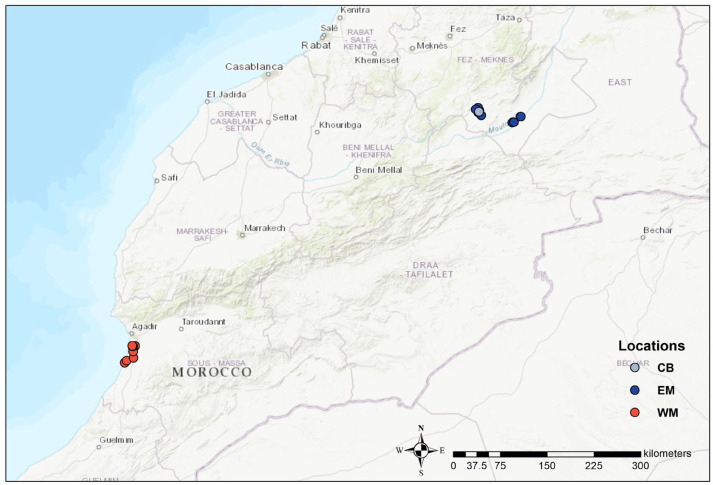
Sampling locations for the genetic assessment of the Emirates Centre for Wildlife Propagation’ Eurasian stone-curlew Conservation Breeding Program in Morocco. CB: Captive-bred individuals sampled at ECWP’s facilities in Enjil; EM: wild individuals sampled in Eastern Morocco; WM: wild individuals sampled in Western Morocco.

We sampled feathers and blood from 87 individuals of the North African subspecies *B. o. saharae* (Table 1). Captive-bred individuals were sampled at ECWP’s facilities in Enjil (hereafter referred to as CB), randomly selected, and presumably unrelated. Wild individuals were sampled during the breeding season (spring/summer) over different years and in two locations: in Western Morocco in 2018 and 2021 (thereafter referred to as WM) and in Eastern Morocco in 2020 (hereafter referred to as EM) (Figure 1). Samples were preserved in absolute ethanol and DNA extracted by Blood & Tissue Kit (QIAGEN^®^, Hilden, Germany) following the manufacturer’s recommended protocol. Sex was identified by genetic methods following the protocol of [43] (Table 1).

### 2.2. Mitochondrial DNA

We amplified a 652 bp fragment of the NADH2 dehydrogenase using primers L5216 and H6313 [44]. PCR-reaction conditions were described previously [45]. We ran PCR products on a 1.5% agarose gel and were purified by ExoSAP-IT (Amersham Biosciences Piscataway, NJ, USA) and sequenced with an ABI SeqStudio Genetic Analyzer. Electropherograms were visualized with CHROMAS 1.45 (http://www.technelysium.com.au, URL (last access: 1 August 2024)). We manually corrected and aligned all sequences using CLUSTALX [46] and used the following steps to determine if sequences were nuclear (NUMTs, [47,48]) or mitochondrial copies. We first checked all sequence chromatograms for double signals. Next, we inspected coding sequence alignments for frameshift mutations and/or stop codons. Finally, we compared the corrected sequences to those in the NCBI database using BLASTX and BLASTN (http://www.ncbi.nlm.nih.gov/BLAST/, URL (last access: 1 August 2024)). We uploaded all haplotypes to the NCBI database (Table S1). GenBank accession numbers are reported in Table S1.

We calculated the number of haplotypes (Nh), haplotypic diversity (h), and nucleotide diversity (π) for each sample using DnaSP v.6.5 [49]. We estimated private allele richness after rarefaction to overcome differences in sample sizes with HP-RARE 1.0 [50]. We used ARLEQUIN v.3.5 [51] to characterize the genetic differentiation among samples using Φ*_ST_*. We constructed a phylogeographic network to determine relationships and relative frequencies in each sample using a Minimum Spanning Network [52] as implemented in PopART 1.7 [53]. We used DnaSP v.6.5 to compute mismatch distribution plots from frequencies of the observed number of nucleotide differences for all pairs of individuals. A multimodal distribution is expected for a population that has maintained a long-term, stable, effective population size. Multimodality reflects the stochastic nature of gene trees under neutral evolution at a stable demographic equilibrium. Although a mismatch plot is convenient for interpreting the demographic history, there are limited statistics for testing hypotheses about its shape, and most of these simply test a null hypothesis of unimodality. Consequently, we considered the raggedness index (rg) and the mean absolute error (MAE) computed with DnaSp. Raggedness [54] is a measure of the smoothness of the distribution, and its value is expected to decrease with the increased flatness expected under a hypothesis of expansion, while positive values reflect equilibrium. Its confidence intervals were provided by computer simulations using the coalescent algorithm in DnaSP. The MAE describes the difference between the observed mismatch distribution and that generated from a simulated expansion event. Like rg, MAE is expected to decrease with an increased probability of expansion. Furthermore, we used DnaSP to investigate demographic history by Tajima’s D [55], Fu and Li’s F [56], and Fu’s Fs [57] tests. Significant negative D, F, and Fs values were considered indicative of a population expansion (following a bottleneck or a selective sweep) or purifying selection.

### 2.3. Microsatellites

#### 2.3.1. Genetic Variability

A total of 22 autosomal microsatellites were used: 18 specifically developed for the Eurasian stone-curlew (BOE series Table S2; [58]) and four obtained, through heterologous amplifications, for a wide range of birds (TG0 series Table S2; [59]. We amplified these markers in multiplex reactions using QIAGEN Multiplex PCR following the manufacturer standard protocol with an annealing temperature (Ta) of 57 °C. We analyzed PCR products with Genemapper v.3.7 (Applied Biosystems Inc., Foster City, CA, USA). We controlled for genotyping errors with multiple blind runs of 7% of individuals [60] to confirm an error rate lower than 0.8–1% [61]. We also checked for scoring errors and large allele dropouts using Microchecker v.2.2.3 [62]. We used Cervus v.3.0 [63] to estimate polymorphic information content and the frequency of null alleles for each locus in each sample. We used 0.2 as a threshold above which the frequency of a null allele is considered high [64,65,66], especially considering the large number of markers used here [67].

We performed all subsequent analyses using R v.4.2.1 [68]. We used hierfstat v.0.5.11 [69] to compute the number of alleles, allelic richness, observed and expected heterozygosity values as well as *F*_IS_ values. We used Genepop v.1.1.7 [70] to evaluate departures from Hardy–Weinberg equilibrium (excess and deficit tests), linkage disequilibrium, population F-statistics, and genic and genotypic differentiations. All tests were performed at the population level with Genepop using 10,000 dememorizations, 1000 batches, and 10,000 iterations per batch. For all multiple comparisons, levels of significance were corrected for multiple tests using the Benjamini–Yekutieli [71] technique [72,73] using the p.adjust of the stats v.4.2.1 package [68]. The level of significance was considered at 0.05.

#### 2.3.2. Relatedness Between Captive Individuals

We used related v.1.0 [74] to compute the Queller and Goodnight relatedness R [75] among captive and wild individuals, assuming no previous inbreeding and employing population-specific allele frequencies. We used relatedness methods since they make no a priori assumption on kinship levels and provide a continuous value of R. A relatedness close to 0 should be obtained for dyads of unrelated individuals, 0.25 for half-sibs, and 0.5 for full sibs [75]. To compare the mean relatedness between groups, we determined the mean values and bootstrapped 95% confidence intervals. Specifically, we performed the bootstrap sampling using the boot v.1.3–28 package, and two-sided non-parametric confidence intervals for the means were generated using the ‘boot.ci’ function with 1000 resamples for each group [76,77].

#### 2.3.3. Analyses of Population Structure and Admixture

We used adegenet v.2.1.8 [78] to carry out PCA analyses, and the ‘find.clusters’ function and Bayesian Information Criterion (BIC) to estimate K. Discriminant analysis of principal components (i.e., DAPC), a method that aims at maximizing variation between samples while minimizing variation within sample, was conducted with the package adegenet v.2.1.8 [78]. We estimated the appropriate number of principal components (PCs) through cross-validation replicates on a training dataset containing 90% of the individuals randomly chosen. Because of significant variations in results with the default value (i.e., rep. = 30), a total of 10,000 replicates were performed for each level of PC retention [79]. The optimal number of principal components retained in our analyses was evaluated as 45, which was associated with the lowest root mean squared error (RMSE). We investigated admixture levels using LEA v.3.8.0 [80]. Computations were performed for K = 1–5 ancestral populations, with 1000 replications per value of K. Values of the cross-entropy criterion for each K were plotted to identify K.

## 3. Results

### 3.1. Mitochondrial DNA

**Figure 2:**
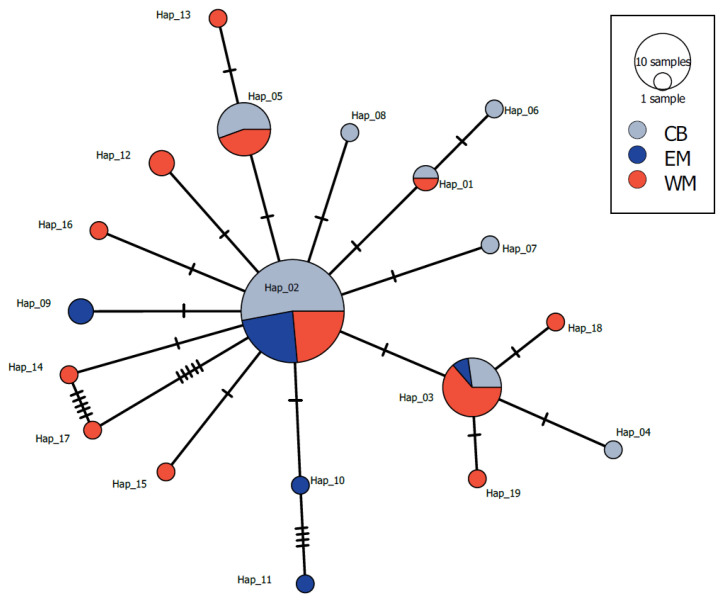
Minimum Spanning Network of haplotypes identified in three samples of Eurasian stone-curlew. The pie chart indicates samples in which the haplotype is found, whereas the size of the pie segments is proportional to the haplotype frequency in the dataset. CB: Captive Bred individuals sampled at ECWP’s facilities in Enjil; EM: wild individuals sampled in Eastern Morocco; WM: wild individuals sampled in Western Morocco.

We successfully retrieved 652-bp mtDNA sequences from 31 captive and 42 wild individuals. We found 22 polymorphic sites, 6 parsimony informative sites, and 6 singleton variable sites; these polymorphic sites identified 19 haplotypes (Table S1, Figure 2). In the minimum-spanning network (Figure 2), the most common haplotypes were shared by both captive and wild samples. All haplotypes together constituted a single haplogroup, with private haplotypes present in both captive and wild samples. Genetic diversity indexes were comparable among captive and eastern Morocco samples, whereas Western Morocco exhibited the highest values of genetic diversity (Table 2). Private allele richness after rarefaction showed values higher in wild samples compared to the captive ones. There was no significant genetic differentiation between samples (Table 3).

Raggedness values were low but not significant for any population. A bimodal distribution was observed for all wild populations in the mismatch (Figures S1–S3). However, the demographic tests showed significant D, F, and Fs values for the population WM (Table 4), which can be a marker of a recent expansion.

### 3.2. Microsatellites

We obtained genotypes from 32 captive and 55 wild individuals. All loci exhibited an estimated frequency of null alleles below the threshold of 0.2. The number of alleles ranged from 4 to 20, the polymorphic information content ranged from 0.137 (BOE01) to 909 (BOE04) (Table S2).

Genetic diversity measures were similar between samples (Table 5) with an allelic richness ranging from 6.35 (CB) to 6.54 (EM) and observed heterozygosities ranging from 0.695 (WM) to 0.705 (CB). F_IS_ values were low (ranging from −0.039 to 0.004); none of the heterozygote excess or deficit tests were statistically significant.

**Figure 3:**
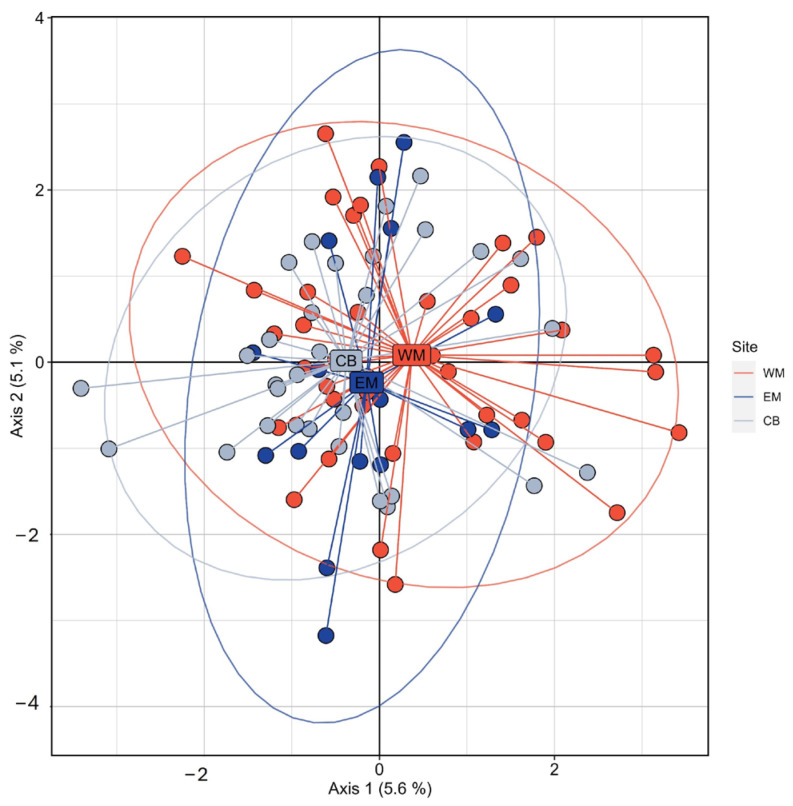
Plots of the first two Principal Components calculated on microsatellite data collected on the three samples of Eurasian stone-curlew. CB: Captive Bred individuals sampled at ECWP’s facilities in Enjil; EM: wild individuals sampled in Eastern Morocco; WM: wild individuals sampled in Western Morocco.

**Figure 4:**
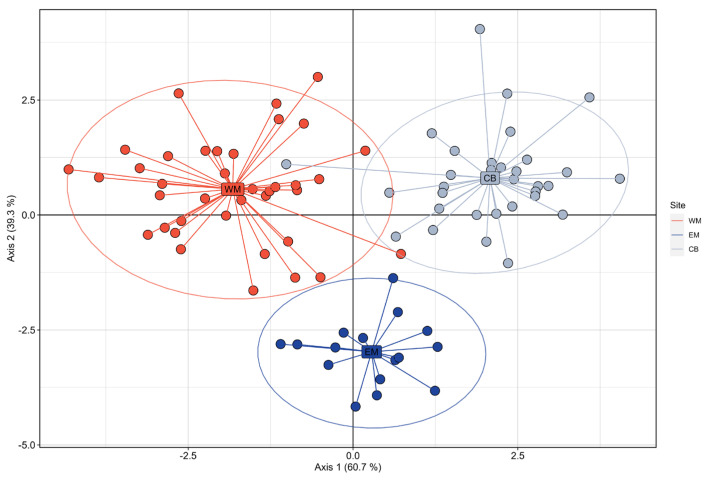
Scatter plot of the first of axes of the Discriminant Analysis after Principal Components (DAPC) calculated on microsatellites data collected on the three samples of Eurasian stone-curlew. CB: Captive Bred individuals sampled at ECWP’s facilities in Enjil; EM: wild individuals sampled in Eastern Morocco; WM: wild individuals sampled in Western Morocco.

F_ST_ values ranged from −0.0078 to 0.0102 and were significant between the captive and wild samples with both genic and genotypic differentiations (Table 3 and Table S3). Both the ‘find.clusters’ function and admixture analyses with LEA provided congruent results with all individuals belonging to a single cluster (i.e., K = 1, Figure S4), and PCA analyses did not result in significant clustering of individuals at a given location (Figure 3). DAPC, however, did identify three subgroups matching each sample (Figure 4).

Relatedness values are very low with mean relatedness ranging from 0.013 within CB to −0.017 in EM (Table 6). Except for the captive individuals, 95% confidence intervals of each group encompass 0, nevertheless, the lower limit for the captive sample was 0.002. Furthermore, confidence intervals computed from bootstrapped data overlapped between all samples analyzed (Table 6) and did not identify any differences in mean relatedness between captive and wild samples.

## 4. Discussion

Our results include multiple genetic analyses carried out on mitochondrial DNA and microsatellites of Eurasian stone-curlew populations in Morocco, a captive bred (CB) and two wild ones, from South-western Morocco close to Agadir (WM) and Eastern Morocco near Enjil (EM). The objective was to investigate the captive flock’s genetic characteristics and compare them to those in the wild. The lack of mitochondrial genetic differentiation between captive and wild samples, and the results of PCA and admixture analyses on microsatellite data indicated no significant clustering. Conversely, pairwise *F*_ST_ and DAPC results from microsatellite data were concordant and exhibited low but significant differences between captive and wild samples. These results were supported by a large number of markers, thus compensating for the relatively reduced number of individual samples in Western Morocco [81,82,83,84]. *F*_ST_ values, as measured with microsatellite markers, between the captive and all wild samples did not exceed 0.01, a level considered to be an indicator of a very low genetic differentiation [85,86]. Very low but significant *F*_ST_ levels could result from changes in allelic frequencies during the captive phase or reflect a scenario of isolation by distance. Discriminant Analysis of Principal Components (DAPC) aims at maximizing variation between samples while minimizing variation within samples; thus, it relies on the maximum discrimination of individuals into groups. In our case, and with such low genetic differentiation, over-discrimination sample populations may have occurred, translating small-scale genetic structures and/or some level of genetic drift in captivity [9,87,88].

Genetic diversity levels between wild and captive samples were investigated with both mtDNA and microsatellite markers. Private allele richness of NADH2 haplotypes in the captive flock was smaller than in wild samples, whilst haplotype and nucleotide diversity were comparable. In addition, mtDNA analyses suggest that wild populations in Western Morocco are showing a recent demographic expansion, a result congruent with a recent field census study in that area that suggests an increase in individuals over a four-year period [89]. D, F, and Fs negative significant values indicate population structure with historical changes, possibly involving expansion only for WM, even though distribution was multimodal. However, a demographic interpretation of the neutrality tests should be applied with caution for organisms that exhibit high population handlings and a small number of breeders or with high variance in reproductive success among breeders. Population dynamics in such cases can deviate significantly from the underlying assumptions of the Wright–Fisher model and Kingman’s coalescent theory [90]. Such population dynamics are then better described by multiple-merger coalescent models [91], and a standard coalescence model would not translate efficiently recent demographic changes [92,93]. Thus, a more extensive and prolonged demographic survey would be required to investigate further the occurrence of potential demographic changes, and the scope of the conservation program for this population will need to be reevaluated to adjust the reinforcement strategy accordingly. In addition, microsatellite markers indicated similar levels of genetic diversity between all samples, with allelic richness of about 5.4 and observed heterozygosities of about 0.7. Heterozygosity values were also similar to the previous ones reported for other wild populations of the species throughout its range [45]. Observed levels of genetic diversity were similar to other birds’ conservation breeding programs, such as the Siberian Crane (*Grus leucogeranus)* [94], Hume’s pheasant (*Syrmaticus humiae*) [95], or the houbara bustard (*Chlamydotis undulata undulata*) [8]. Furthermore, no significant heterozygote deficit was identified through *F*_IS_ analyses, which could have suggested a potential Wahlund effect where an analyzed sample is composed of a mix of individuals from different origins [96,97]. *F*_IS_ values were marginal in all samples, indicating the absence of any inbreeding. Mean relatedness was not significantly different from 0 within wild samples, and the captive flock presented a slightly higher mean relatedness, although it was very small (i.e., 0.013). That can be expected within a captive/closed population [9,10]; nevertheless, the magnitude of that difference was marginal. Finally, the overlap of confidence intervals between groups indicates that there is no significant difference in average relatedness between each sample; captive-bred individuals are not highly related compared to their wild counterparts.

Strict genetic management through pedigree analyses and pairing selection would ensure both inbreeding avoidance and maintenance of genetic diversity within the captive flock [8,10,12]. Nevertheless, the captive flock might have experienced some level of genetic drift, and we recommend implementing regular additions of a limited number of wild individuals to circumvent this. In houbara bustard (*Chlamydotis undulata undulata*), such an approach of combining strict genetic management of the captive flock with the regular addition of founders proves successful in maintaining genetic diversity in captivity while efficiently preventing both inbreeding and adaptation to captivity [8,14].

Finally, the addition of post-release movement studies will deliver valuable insight into the dispersion and settlement of captive-bred individuals, thus providing crucial information for the implementation of a holistic and efficient conservation strategy for the Eurasian stone-curlew in Morocco as part of a One Plan approach where both wild and captive compartments are integrated into the genetic management strategy to optimize the conservation outcomes [1,2,98].

## 5. Conclusions

It is crucial for a conservation breeding program supporting reinforcement measures for its founders to be both representative of genetic diversity in the wild and closely related to recipient populations. Here, we confirmed that the captive flock of Eurasian stone-curlews at ECWP showed very low genetic differentiation levels from wild populations, thus confirming the Moroccan origin of the founders. On a broader scale, and with an increasing number of translocation programs using captive-bred individuals as a source for translocations, this study highlights essential processes that should be implemented to ensure effective genetic management of the captive source, preventing both the loss of genetic diversity and adaptation to captivity.

## Figures and Tables

**Table 1:** Samples used to compare captive and wild individuals of Eurasian stone-curlew in Morocco. For each sample, the origin of individuals (i.e., captive-bred or wild), the sampling site, the acronym used throughout the document, the total sample size as well as the sample size used with each type of molecular marker are reported. The number of males and females are reported in that order brackets for each sample (i.e., males, females).

Group	Sampling Area	Acronym	Total Sample Size	Mitochondrial Sample Size	Microsatellite Sample Size
Captive-bred	ECWP	CB	32 (16, 16)	31 (15, 16)	32 (16, 16)
Wild	Western Morocco	WM	39 (22, 17)	29 (16, 13)	39 (22, 17)
	Eastern Morocco	EM	16 (5, 11)	13 (4, 9)	16 (5, 11)
Total			87 (43, 44)	73 (36, 37)	87 (43, 44)

**Table 2:** Polymorphism for the mitochondrial NADH2 region (652 bp) measured for three samples of Eurasian stone-curlew. Number of individuals per sample (N), number of haplotypes (Nh), number of private haplotypes (NhaP), haplotype diversity (H), nucleotide diversity (π) and its standard deviation, and private allele richness after rarefaction (PAr) for each sample.

Group	Sampling Location	N	Nh	NhaP	H	π	P_Ar_
Captive-bred	ECWP (CB)	31	8	4	0.643	0.0014 (0.0003)	5.07
Wild	Western Morocco (WM)	29	12	8	0.862	0.0026 (0.0005)	7.50
	Eastern Morocco (EM)	13	5	3	0.628	0.0020 (0.0009)	4.57

**Table 3:** Results from genetic differentiation analyses between three samples of Eurasian stone-curlew. Pairwise *Φ*_ST_ values using the mitochondrial DNA NADH2 region (652 bp) are presented above the diagonal. Pairwise *F*_ST_ values and associated exact test of genetic differentiation using 22 microsatellite loci are presented below diagonal. CB: Captive Bred individuals sampled at ECWP’s facilities in Enjil; EM: wild individuals sampled in Eastern Morocco; WM: wild individuals sampled in Western Morocco. Significance (*p* < 0.05) after the Benjamini-Yekutieli correction is indicated by *.

	CB	EM	WM
CB		−0.0011	0.0517
EM	0.0078 *		0.0720
WM	0.0098 *	0.0102 *	

**Table 4:** Results of demographic inferences through mitochondrial tests using rg (raggedness) MAE (Mean Absolute Error), Tajima’s D, Fu and Li’s F, and Fu’s Fs tests, as well as Mismatch Distribution (MD). Significance (*p* < 0.05) is indicated by *.

Group	Sampling Location	Rg	MAE	F	FS	D	MD
Captive-bred	ECWP (CB)	0.095	0.471	−1.537	−1.634	−1.435	Unimodal
Wild	Western Morocco (WM)	0.133	0.696	−3.026 *	−2.828 *	−1.889 *	Multimodal
	Eastern Morocco (EM)	0.139	0.491	−1.402	−1.495	−1.551	Multimodal

**Table 5:** Summary statistics computed from 22 microsatellite loci among three samples of Eurasian stone-curlew. Sample size (N), Na: average number of alleles per locus and per sample, KAR: allelic richness, heterozygosity (He: expected and Ho: observed), F_IS_: inbreeding coefficient, *p* values for the Hardy-Weinberg disequilibrium exact test (excess and deficit tests). CB: Captive Bred individuals sampled at ECWP’s facilities in Enjil; EM: wild individuals sampled in Eastern Morocco; WM: wild individuals sampled in Western Morocco.

Sampling Location	N	N_a_	K_AR_	H_o_	H_e_	F_IS_	Heterozygote Deficit Test	Heterozygote Excess Test
CB	32	7.59	6.35	0.705	0.679	−0.039	0.389	0.6103
WM	39	7.86	6.34	0.695	0.69	−0.007	0.497	0.526
EM	16	6.68	6.54	0.697	0.699	0.004	0.474	0.504

**Table 6:** Queller and Goodnight (1989) relatedness estimates using 22 microsatellite loci for three samples of Eurasian stone-curlew. The sample size refers to the number of dyads analyzed. Means are presented, along with their standard deviation and median values. A thousand bootstrap replicates were generated to compute 95% confidence intervals (CI) of average relatedness per group; lower and upper limits are presented. CB: Captive Bred individuals sampled at ECWP’s facilities in Enjil; EM: wild individuals sampled in Eastern Morocco; WM: wild individuals sampled in Western Morocco.

Sampling Location	Sample Size	Mean	Standard Deviation	Median	CI (Lower Limit)	CI (Upper Limit)
CB	496	0.013	0.132	0.007	0.002	0.025
WM	741	−0.004	0.131	−0.01	−0.012	0.007
EM	120	−0.017	0.131	−0.034	−0.044	0.007

## Data Availability

The data that support the findings of this study are available upon reasonable request from the corresponding author. The data are not publicly available due to privacy or ethical restrictions.

## Supplementary Materials

The following supporting information can be downloaded at https://www.mdpi.com/article/10.3390/biology13120982/s1, Table S1: haplotype diversity; Table S2: microsatellite diversity per locus; Table S3: microsatellite genetic differentiation; Figure S1: Mismatch distribution plots from frequencies of observed number of nucleotide differences for all pairs of individuals within the captive bred population (CB); Figure S2: Mismatch distribution plots from frequencies of observed number of nucleotide differences for all pairs of individuals within the eastern Morocco population (EM).; Figure S3: Mismatch distribution plots from frequencies of observed number of nucleotide differences for all pairs of individuals within the western Morocco population (WM); Figure S4: Cross entropy plot. Computations were performed for K = 1-5 ancestral populations, with 1000 replications per value of K.

## References

[1] Byers O., Lees C., Wilcken J., Schwitzer C. (2013). The One Plan Approach: The Philosophy and Implementation of CBSG’s Approach to Integrated Species Conservation Planning. WAZA Mag..

[2] Traylor-Holzer K., Leus K., Bauman K. (2019). Integrated Collection Assessment and Planning (ICAP) workshop: Helping zoos move toward the One Plan Approach. Zoo Biol..

[3] Pizzutto C.S., Colbachini H., Jorge-Neto P.N. (2021). One Conservation: The integrated view of biodiversity conservation. Anim. Reprod..

[4] Conde D.A., Flesness N., Colchero F., Jones O.R., Scheuerlein A. (2011). Conservation. An emerging role of zoos to conserve biodiversity. Science.

[5] Pritchard D.J., Fa J.E., Oldfield S., Harrop S.R. (2012). Bring the captive closer to the wild: Redefining the role of ex-situ conservation. Oryx.

[6] IUCN Species Survival Commission (SSC) (2014). IUCN Species Survival Commission Guidelines on the Use of Ex-Situ Management for Species Conservation.

[7] Galla S.J., Moraga R., Brown L., Cleland S., Hoeppner M.P., Maloney R.F., Richardson A., Slater L., Santure A.W., Steeves T.E. (2020). A comparison of pedigree, genetic and genomic estimates of relatedness for informing pairing decisions in two critically endangered birds: Implications for conservation breeding programmes worldwide. Evol. Appl..

[8] Rabier R., Robert A., Lacroix F., Lesobre L. (2020). Genetic assessment of a conservation breeding program of the Houbara bustard (*Chlamydotis undulata undulata*) in Morocco, based on pedigree and molecular analyses. Zoo Biol..

[9] Lacy R.C. (1987). Loss of Genetic Diversity from Managed Populations: Interacting Effects of Drift, Mutation, Immigration, Selection, and Population Subdivision. Conserv. Biol..

[10] Lacy R.C., Bowles M.L., Whelan C.J. (1994). Managing genetic diversity in captive populations of animals. Restoration of Endangered Species: Conceptual Issues, Planning, and Implementation.

[11] Ballou J.D., Lacy R.C., Ballou J.D., Foose T.J., Gilpin M.E. (1995). Identifying genetically important individuals for management of genetic variation in pedigreed populations. Population Management for Survival and Recovery: Analytical Methods and Strategies in Small Population Conservation.

[12] Ballou J.D., Foose T.J., Kleiman D.G., Allen M.E., Thompson K.V., Lumpkin S. (1996). Demographic and Genetic Management of Captive Populations. Wild Mammals in Captivity: Principles and Techniques.

[13] Witzenberger K.A., Hochkirch A. (2011). Ex-situ conservation genetics: A review of molecular studies on the genetic consequences of captive breeding programmes for endangered animal species. Biodivers. Conserv..

[14] Rabier R., Lesobre L., Robert A. (2021). Reproductive performance in houbara bustard is affected by the combined effects of age, inbreeding and number of generations in captivity. Sci. Rep..

[15] Soulé M.E., Soulé M.E., Wilcox B.A. (1980). Thresholds for survival: Maintaining fitness and evolutionary potential. Conservation Biology: An Evolutionary-Ecological Perspective.

[16] Ballou J.D., Foose T.J., Gilpin M.E. (1995). Population Management for Survival and Recovery: Analytical Methods and Strategies in Small Population Conservation.

[17] Hedrick P.W., Kalinowski S.T. (2000). Inbreeding Depression in Conservation Biology. Annu. Rev. Ecol. Syst..

[18] Frankham R. (2008). Genetic adaptation to captivity in species conservation programs. Mol. Ecol..

[19] Robert A. (2009). Captive breeding genetics and reintroduction success. Biol. Conserv..

[20] Olden J.D., Leroy Poff N., Douglas M.R., Douglas M.E., Fausch K.D. (2004). Ecological and evolutionary consequences of biotic homogenization. Trends Ecol. Evol..

[21] Volis S., Blecher M. (2010). Quasi in-situ: A bridge between ex-situ and in-situ conservation of plants. Biodivers. Conserv..

[22] IUCN Species Survival Commission (SSC) (2013). Guidelines for Reintroductions and Other Conservation Translocations Version 1.0.

[23] Lacy R.C., Ballou J.D., Princée F.P., Starfield A., Thompson E.A., Ballou J.D., Foose T.J., Gilpin M.E. (1995). Pedigree analysis for population management. Population Management for Survival and Recovery: Analytical Methods and Strategies in Small Population Conservation.

[24] Hogg C.J., Wright B., Morris K.M., Lee A.V., Ivy J.A., Grueber C.E., Belov K. (2019). Founder relationships and conservation management: Empirical kinships reveal the effect on breeding programmes when founders are assumed to be unrelated. Anim. Conserv..

[25] Rabier R., Erlichman A., Lesobre L., Robert A. (2022). The necessity of considering founder kinships in conservation breeding programs. Anim. Conserv..

[26] Hume R., Kirwan G.M., del Hoyo J., Elliott A., Sargatal J., Christie D.A., de Juana E. (2019). Eurasian Thick-knee (*Burhinus oedicnemus*). Handbook of the Birds of the World Alive.

[27] Cremene C., Groza G., Rakosy L., Schileyko A.A., Baur A., Erhardt A., Baur B. (2005). Alterations of Steppe-Like Grasslands in Eastern Europe: A Threat to Regional Biodiversity Hotspots. Conserv. Biol..

[28] Santos T., Suarez F., Bota G. (2005). Biogeography and population trends of Iberian steppe birds. Ecology and Conservation of Steppe-Land Birds.

[29] Birdlife International (2018). State of the World’s Birds: Taking the Pulse of the Planet.

[30] Delany S., Scott D., Dodman T., Stroud D. (2009). An Atlas of Wader Populations in Africa and Western Eurasia.

[31] Triolo S., Campobello D., Sarà M. (2011). Diurnal habitat suitability for a Mediterranean steppeland bird, identified by Ecological Niche Factor Analysis. Wildl. Res..

[32] Cramp S., Brooks D.J. (1983). Handbook of the Birds of Europe, the Middle East and North Africa: The Birds of the Western Palearctic.

[33] Teyar Y., Giunchi D., Baratti M., Falchi V., Znari M., Aourir M. (2020). Does the Breeding Biology of the Eurasian Stone-Curlew *Burhinus oedicnemus* in South-Western Morocco Differ between Grazed Steppe and Irrigated Farmland?. Acta Ornithol..

[34] Dengler J., Janišová M., Török P., Wellstein C. (2014). Biodiversity of Palaearctic grasslands: A synthesis. Agric. Ecosyst. Environ..

[35] Kusi K.K., Khattabi A., Mhammdi N. (2022). Analyzing the impact of land use change on ecosystem service value in the main watersheds of Morocco. Environ. Dev. Sustain..

[36] Platt J.B. (1984). Falcon breeding as a conservation tool in Arabia. Int. Zoo Yearb..

[37] Clark T. (2004). The noble art of the chase in the Arab world. Asian Aff..

[38] Kittelberger K.D., Buechley E.R., Ford M., Ağırkaya K., Hakkı Şekercioğlu Ç. (2021). First satellite-tracked migration of an Eurasian Thick-knee (*Burhinus oedicnemus*) in the Middle East ends in human-caused mortality. Zool. Middle East.

[39] International Fund for Houbara Conservation Website. https://houbarafund.gov.ae.

[40] Montgomery M.E., Ballou J.D., Nurthen R.K., England P.R., Briscoe D.A., Frankham R. (1997). Minimizing kinship in captive breeding programs. Zoo Biol..

[41] Giglio R.M., Ivy J.A., Jones L.C., Latch E.K. (2016). Evaluation of alternative management strategies for maintenance of genetic variation in wildlife populations. Anim. Conserv..

[42] Allendorf F. (1993). Delay of Adaptation to Captive Breeding by Equalizing Family Size. Conserv. Biol..

[43] Griffiths R.A., Double M.C., Orr K., Dawson R.J.G. (1998). A DNA test to sex most birds. Mol. Ecol..

[44] Sorenson M.D., Ast J.C., Dimcheff D.E., Yuri T., Mindell D.P. (1999). Primers for a PCR-based approach to mitochondrial genome sequencing in birds and other vertebrates. Mol. Phylogenet. Evol..

[45] Mori A., Giunchi D., Rodríguez-Godoy F., Grasso R., Baldaccini N.E., Baratti M. (2017). Multilocus approach reveals an incipient differentiation process in the Stone-curlew, *Burhinus oedicnemus* around the Mediterranean basin. Conserv. Genet..

[46] Thompson J.D., Gibson T.J., Des Higgins G. (2002). Multiple sequence alignment using ClustalW and ClustalX. Curr. Protoc. Bioinform..

[47] Song H., Buhay J.E., Whiting M.F., Crandall K.A. (2008). Many species in one: DNA barcoding overestimates the number of species when nuclear mitochondrial pseudogenes are coamplified. Proc. Natl. Acad. Sci. USA.

[48] Buhay J.E. (2009). “COI-like” Sequences Are Becoming Problematic in Molecular Systematic and DNA Barcoding Studies. J. Crustac. Biol..

[49] Rozas J., Ferrer-Mata A., Sánchez-DelBarrio J.C., Guirao-Rico S., Librado P., Ramos-Onsins S.E., Sánchez-Gracia A. (2017). DnaSP 6: DNA Sequence Polymorphism Analysis of Large Data Sets. Mol. Biol. Evol..

[50] Kalinowski S.T. (2005). hp-rare 1.0: A computer program for performing rarefaction on measures of allelic richness. Mol. Ecol. Notes.

[51] Excoffier L., Lischer H.E.L. (2010). Arlequin suite ver 3.5: A new series of programs to perform population genetics analyses under Linux and Windows. Mol. Ecol. Resour..

[52] Bandelt H.J., Forster P., Röhl A. (1999). Median-joining networks for inferring intraspecific phylogenies. Mol. Biol. Evol..

[53] Leigh J.W., Bryant D. (2015). POPART: Full-feature software for haplotype network construction. Methods Ecol. Evol..

[54] Harpending H.C. (1994). Signature of ancient population growth in a low-resolution mitochondrial DNA mismatch distribution. Hum. Biol..

[55] Tajima F. (1989). Statistical method for testing the neutral mutation hypothesis by DNA polymorphism. Genetics.

[56] Fu Y.X., Li W.H. (1993). Statistical tests of neutrality of mutations. Genetics.

[57] Fu Y.X. (1997). Statistical tests of neutrality of mutations against population growth, hitchhiking and background selection. Genetics.

[58] Mori A., Dawson D.A., Horsburgh G.J., Giunchi D., Baldaccini N.E., Baratti M. (2014). Characterisation of microsatellite markers in the stone curlew *Burhinus oedicnemus*. Conserv. Genet. Resour..

[59] Dawson D.A., Horsburgh G.J., Küpper C., Stewart I.R.K., Ball A.D., Durrant K.L., Hansson B., Bacon I., Bird S., Klein A. (2010). New methods to identify conserved microsatellite loci and develop primer sets of high cross-species utility—As demonstrated for birds. Mol. Ecol. Resour..

[60] Pompanon F., Bonin A., Bellemain E., Taberlet P. (2005). Genotyping errors: Causes, consequences and solutions. Nat. Rev. Genet..

[61] Bonin A., Bellemain E., Bronken Eidesen P., Pompanon F., Brochmann C., Taberlet P. (2004). How to track and assess genotyping errors in population genetics studies. Mol. Ecol..

[62] Van Oosterhout C., Hutchinson W.F., Wills D.P.M., Shipley P. (2004). micro-checker: Software for identifying and correcting genotyping errors in microsatellite data. Mol. Ecol. Notes.

[63] Kalinowski S.T., Taper M.L., Marshall T.C. (2007). Revising how the computer program CERVUS accommodates genotyping error increases success in paternity assignment. Mol. Ecol..

[64] Dakin E.E., Avise J.C. (2004). Microsatellite null alleles in parentage analysis. Heredity.

[65] Chapuis M.-P., Estoup A. (2007). Microsatellite null alleles and estimation of population differentiation. Mol. Biol. Evol..

[66] Carlsson J. (2008). Effects of microsatellite null alleles on assignment testing. J. Hered..

[67] Wagner A.P., Creel S., Kalinowski S.T. (2006). Estimating relatedness and relationships using microsatellite loci with null alleles. Heredity.

[68] R Core Team (2022). R: A Language and Environment for Statistical.

[69] Goudet J. (2005). hierfstat, a package for r to compute and test hierarchical F-statistics. Mol. Ecol. Notes.

[70] Rousset F. (2008). genepop’007: A complete re-implementation of the genepop software for Windows and Linux. Mol. Ecol. Resour..

[71] Yekutieli D., Benjamini Y. (2001). The Control of the False Discovery Rate in Multiple Testing Under Dependency. Ann. Stat..

[72] Narum S.R. (2006). Beyond Bonferroni: Less conservative analyses for conservation genetics. Conserv. Genet..

[73] White T., van der Ende J., Nichols T.E. (2019). Beyond Bonferroni revisited: Concerns over inflated false positive research findings in the fields of conservation genetics, biology, and medicine. Conserv. Genet..

[74] Pew J., Muir P.H., Wang J., Frasier T.R. (2015). related: An R package for analysing pairwise relatedness from codominant molecular markers. Mol. Ecol. Resour..

[75] Queller D.C., Goodnight K.F. (1989). Estimating relatedness using genetic markers. Evolution.

[76] Davison A.C., Hinkley D. (1997). Bootstrap Methods and Their Application.

[77] Canty A., Ripley B.D. (2022). Boot: Bootstrap R (S-Plus) Functions.

[78] Jombart T. (2008). adegenet: A R package for the multivariate analysis of genetic markers. Bioinformatics.

[79] Jombart T., Collins C. (2015). A Tutorial for Discriminant Analysis of Principal Components (DAPC) Using Adegenet 2.0.0.

[80] Jombart T., Devillard S., Balloux F. (2010). Discriminant analysis of principal components: A new method for the analysis of genetically structured populations. BMC Genet..

[81] Koskinen M.T., Hirvonen H., Landry P.-A., Primmer C.R. (2004). The benefits of increasing the number of microsatellites utilized in genetic population studies: An empirical perspective. Hereditas.

[82] Hale M.L., Burg T.M., Steeves T.E. (2012). Sampling for microsatellite-based population genetic studies: 25 to 30 individuals per population is enough to accurately estimate allele frequencies. PLoS ONE.

[83] Willing E.-M., Dreyer C., van Oosterhout C. (2012). Estimates of Genetic Differentiation Measured by FST Do Not Necessarily Require Large Sample Sizes When Using Many SNP Markers. PLoS ONE.

[84] Hoban S.M., Gaggiotti O.E., Bertorelle G. (2013). The number of markers and samples needed for detecting bottlenecks under realistic scenarios, with and without recovery: A simulation-based study. Mol. Ecol..

[85] Frankham R., Ballou J.D., Briscoe D.A. (2010). Introduction to Conservation Genetics.

[86] Allendorf F.W., Luikart G., Byrne M., Aitken S.N., Funk W.C. (2021). Conservation and the Genomics of Populations.

[87] Gooley R.M., Tamazian G., Castañeda-Rico S., Murphy K.R., Dobrynin P., Ferrie G.M., Haefele H., Maldonado J.E., Wildt D.E., Pukazhenthi B.S. (2020). Comparison of genomic diversity and structure of sable antelope (*Hippotragus niger*) in zoos, conservation centers, and private ranches in North America. Evol. Appl..

[88] Shier D.M., Navarro A.Y., Tobler M., Thomas S.M., King S.N.D., Mullaney C.B., Ryder O.A. (2021). Genetic and ecological evidence of long-term translocation success of the federally endangered Stephens’ kangaroo rat. Conserv. Sci. Pract..

[89] Teyar Y., Giunchi D., El Bekkay M., Oubrou W., Aourir M. (2024). Hivernage de l’Œdicnème criard *Burhinus oedicnemus* sur le littoral atlantique de souss-massa (maroc) : Effectifs et phénologie des rassemblements. Alauda.

[90] Kingman J. (1982). The coalescent. Stoch. Process. Their Appl..

[91] Tellier A., Lemaire C. (2014). Coalescence 2.0: A multiple branching of recent theoretical developments and their applications. Mol. Ecol..

[92] Sargsyan O., Wakeley J. (2008). A coalescent process with simultaneous multiple mergers for approximating the gene genealogies of many marine organisms. Theor. Popul. Biol..

[93] Eldon B., Birkner M., Blath J., Freund F. (2015). Can the site-frequency spectrum distinguish exponential population growth from multiple-merger coalescents?. Genetics.

[94] Mudrik E.A., Kashentseva T.A., Postelnykh K.A., Nosachenko G.V., Politov D.V. (2014). Genetic diversity and relatedness in different generations of the Siberian crane (*Grus leucogeranus* Pallas) captive population. Russ. J. Genet..

[95] Thintip J., Singchat W., Ahmad S.F., Ariyaraphong N., Muangmai N., Chamchumroon W., Pitiwong K., Suksavate W., Duangjai S., Duengkae P. (2021). Reduced genetic variability in a captive-bred population of the endangered Hume’s pheasant (*Syrmaticus humiae*, Hume 1881) revealed by microsatellite genotyping and D-loop sequencing. PLoS ONE.

[96] Wahlund S. (1928). Zusammensetzung von Populationen und Korrelationserscheinungen vom Standpunkt der Vererbungslehre aus Betrachtet. Hereditas.

[97] Rousset F. (2004). Genetic Structure and Selection in Subdivided Populations.

[98] Hoban S., Bruford M.W., Funk W.C., Galbusera P., Griffith M.P., Grueber C.E., Heuertz M., Hunter M.E., Hvilsom C., Stroil B.K. (2021). Global Commitments to Conserving and Monitoring Genetic Diversity Are Now Necessary and Feasible. Bioscience.

