# Supplementary material for: Genetic Assessment of a Captive Population of Eurasian Stone-Curlew (Burhinus oedicnemus), Source for the Reinforcement of Wild Populations
Source: Biology (Basel). 2024 Nov 27;13(12):982. doi: 10.3390/biology13120982 (PMC11726720; doi:10.3390/biology13120982)
Supplement: Supplementary file 1 [file biology-13-00982-s001.zip › biology-3285361-supplementary.pdf]

Table S1. Absence/Presence (0/1) of the 19 haplotypes identified in the studied populations of Eurasian stone-curlew and their Genbank Accession Numbers. CB: Captive Bred individuals sampled at ECWP's facilities in Enjil; EM: wild individuals sampled in Eastern Morocco; WM: wild individuals sampled in Western Morocco.

Haplotypes	CB	EM	WM	Genbank accession number
Morocco 01	1	1	1	ON736841
Morocco 02	1	1	1	ON736842
Morocco 03	1	0	1	ON736843
Morocco 04	1	0	1	ON736844
Morocco 05	1	0	0	ON736845
Morocco 06	1	0	0	ON736846
Morocco 07	1	0	0	ON736847
Morocco 08	1	0	0	ON736848
Morocco 09	0	0	1	ON736849
Morocco 10	0	0	1	ON736850
Morocco 11	0	0	1	ON736851
Morocco 12	0	0	1	ON736852
Morocco 13	0	0	1	ON736853
Morocco 14	0	0	1	ON736854
Morocco 15	0	0	1	ON736855
Morocco 16	0	0	1	ON736856
Morocco 17	0	1	0	ON736857
Morocco 18	0	1	0	ON736858
Morocco 19	0	1	0	ON736859

Table S2. Summary statistics computed for 22 microsatellite loci in three samples of Eurasian stone-curlew. Microsatellites range of sizes, number of alleles, polymorphic information content, observed and expected heterozygosity, F_{is} (inbreeding coefficient/heterozygote deficit) and possible presence of null alleles are reported.

	Range	Number of alleles	Polymorphic information content	Observed heterozygosity	Expected heterozygosity	Fis	Null allele frequency
BOE01	105-126	4	0.137	0.157	0.149	-0.059	-0.030
BOE02	169-189	8	0.689	0.854	0.741	-0.153	-0.078
BOE03	123-135	6	0.549	0.735	0.623	-0.180	-0.088
BOE04	174-230	9	0.608	0.709	0.663	-0.069	-0.056
BOE05	179-225	12	0.819	0.838	0.839	0.000	-0.021
BOE06	145-241	19	0.904	0.902	0.911	0.009	0.009
BOE07	193-229	8	0.769	0.881	0.810	-0.088	-0.046
BOE08	197-237	10	0.758	0.846	0.778	-0.088	-0.052
BOE09	240-316	11	0.855	0.806	0.865	0.068	0.036
BOE11	198-302	19	0.873	0.824	0.879	0.063	0.048
BOE12	207-365	20	0.746	0.685	0.760	0.099	0.060
BOE13	164-204	12	0.748	0.777	0.782	0.006	-0.005
BOE14	348-437	12	0.872	0.860	0.880	0.023	0.006
BOE15	283-311	5	0.499	0.684	0.604	-0.134	-0.080
BOE17	196-297	15	0.891	0.901	0.899	-0.003	-0.003
BOE18	203-235	7	0.793	0.686	0.820	0.164	0.110
BOE19	262-272	5	0.552	0.670	0.603	-0.112	-0.061
BOE20	181-221	17	0.726	0.852	0.765	-0.114	-0.059
TG01000	185-193	4	0.441	0.398	0.509	0.218	0.116
TG03002	120-126	3	0.517	0.549	0.585	0.061	0.017
TG04004	154-160	3	0.206	0.240	0.223	-0.075	-0.011
TG01124	385-393	4	0.416	0.527	0.487	-0.083	-0.039

Table S3. Results from genetic differentiation analyses between three samples of Eurasian stone-curlew. Pairwise FST values and associated exact test of genetic differentiation using 22 microsatellite loci are presented. Genic and genotypic differentiations levels of significance are corrected for multiple tests using the Benjamini–Yekutieli technique. CB: Captive Bred individuals sampled at ECWP's facilities in Enjil; EM: wild individuals sampled in Eastern Morocco; WM: wild individuals sampled in Western Morocco.

Location 1	Location 2	F_{st}	Genic differentiation	Genic differentiation Corrected BY	Genotypic differentiation	Genotypic differentiation Corrected BY
CB	EM	0.0078	0.0005	0.0010	0.0007	0.0010
CB	WM	0.0098	0.0000	0.0000	0.0000	0.0000
EM	WM	0.0102	0.0005	0.0010	0.0006	0.0010

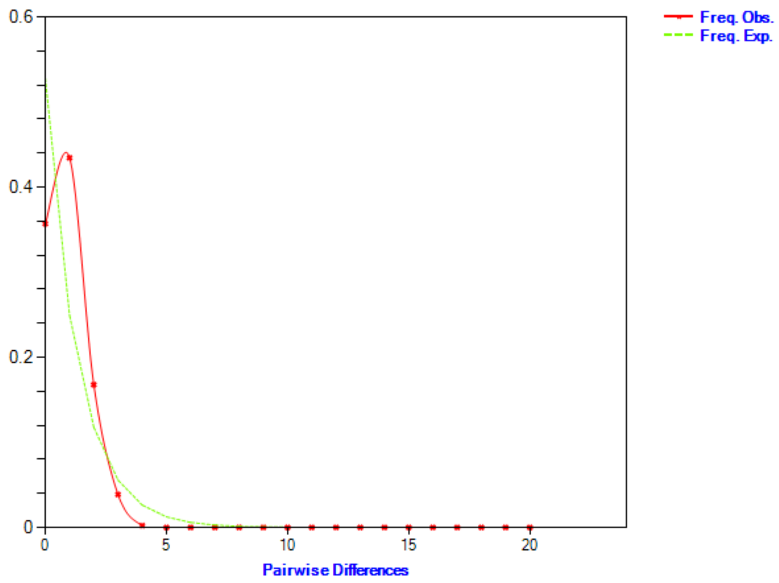

Figure S1. Mismatch distribution plots from frequencies of observed number of nucleotide differences for all pairs of individuals within the captive bred population (CB).

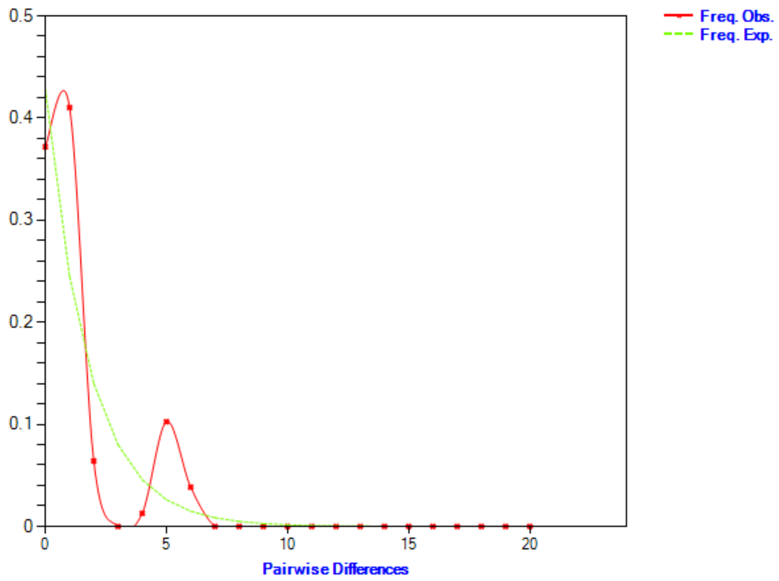

Figure S2. Mismatch distribution plots from frequencies of observed number of nucleotide differences for all pairs of individuals within the eastern Morocco population (EM).

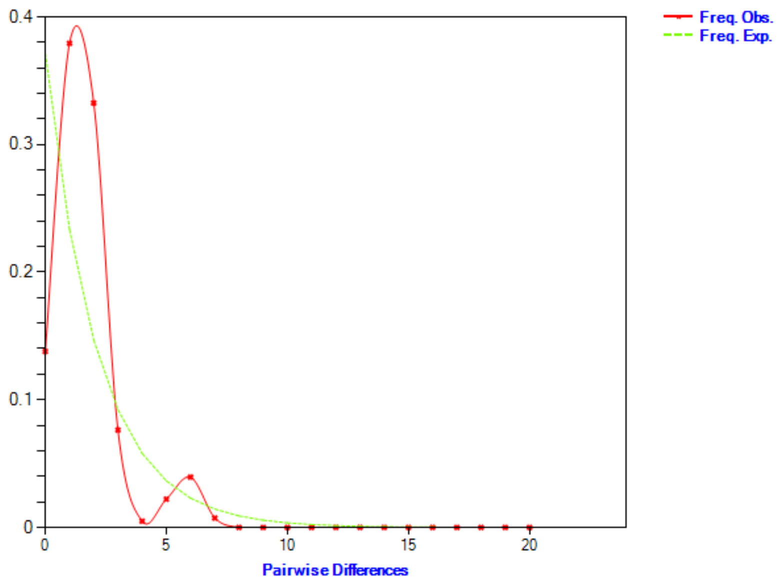

Figure S3. Mismatch distribution plots from frequencies of observed number of nucleotide differences for all pairs of individuals within the western Morocco population (WM).

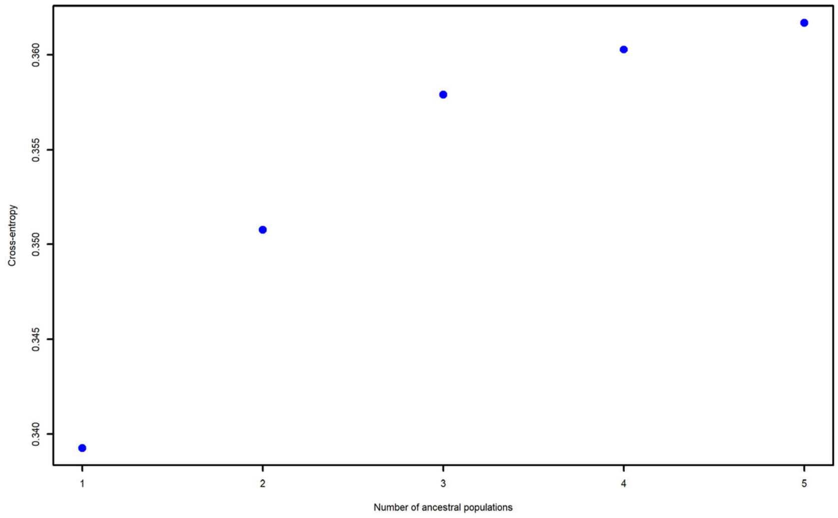

Figure S4. Cross entropy plot. Computations were performed for K = 1-5 ancestral populations, with 1 000 replications per value of K. Values of the cross-entropy criterion for each K was plotted to identify K.
